# Correlation between gonial angle and dynamic tongue collapse in children with snoring/sleep disordered breathing – an exploratory pilot study

**DOI:** 10.1186/s40463-018-0285-8

**Published:** 2018-06-04

**Authors:** S. Anderson, N. Alsufyani, A. Isaac, M. Gazzaz, H. El-Hakim

**Affiliations:** 1 Department of Dentistry, Faculty of Medicine and Dentistry, University of Alberta, Edmonton, Canada; 2 Department of Oral Medicine and Diagnostic Sciences, College of Dentistry, King Saud University Division of Otolaryngology-Head and Neck Surgery Department of Surgery, Riyadh, Saudi Arabia; 3 University of Alberta, Edmonton, AB Canada; 4 Division of Pediatric Surgery, Department of Surgery, Stollery Children’s Hospital, Edmonton, AB Canada; 5 Division of Otolaryngology-Head and Neck Surgery, Department of Surgery, University of Alberta, 2C3.57 Walter MacKenzie Centre, 8440 112 St NW, Edmonton, AB T6G 2R7 Canada

**Keywords:** Pediatric sleep disordered breathing, Maxillo-mandibular disproportion, Gonial angle, Adeno-tonsillectomy, Drug induced sleep endoscopy, Pharyngeal collapse

## Abstract

**Background:**

Drug induced sleep endoscopy (DISE) is hoped to identify reasons of failure of adenotonsillectomy (AT) in treating pediatric sleep disordered breathing (SDB). Maxillomandibular disproportion has been studied as another association which may explain alternative pathogenesis of SDB. We aimed to explore the relation between the size of the gonial angle and inclination of the epiglottis measured from cone beam CT (CBCT) and tongue base collapse based on DISE in children with SDB.

**Method:**

A retrospective chart review was conducted at a tertiary pediatric center. Children (6-17 years old) assessed at a multi-disciplinary Upper Airway Clinic, diagnosed with SDB and maxillo-mandibular disproportion (MMD), and who underwent DISE were eligible. Variables obtained from the electronic medical records of the clinic and prospective database included demographics, comorbidities, surgeries performed, investigations, DISE findings and CBCT findings. The gonial angle of subjects with and without tongue base collapse (TBC) on SNP were compared.

**Results:**

In total 29 patients (13 male, 8 female) age 6-17 (median= 9) were eligible for the study from January 2009 – July 2016. We included 11 subjects, and 10 comparators. The mean gonial angle of the TBC group was 139.3°± 7.6°, while that of the comparison group was 129.4°±3.5 (mean difference -9.937, 95% CI of -15.454 to - 4.421, P = 0.001, power of test 0.95). Additionally, the mean inclination of the epiglottis had a mild positive correlation (r=0.32, *p*<0.05) with the gonial angle, in the whole cohort.

**Conclusions:**

This pilot study suggests that TBC may be mediated by a wider gonial angle in children with SDB patients. The posterior tilt of the epiglottis on CBCT may be a surrogate sign of TBC.

## Background

The American Academy of Otolaryngology – Head and Neck Surgery defines Pediatric sleep disordered breathing (SDB) as difficulty in breathing during sleep, which can range from habitual snoring to obstructive sleep apnea [1]. Current evidence suggests a strong association with multiple negative outcomes which include cardiovascular, metabolic, behavioral and learning consequences, as well as increased rate of nocturnal enuresis [1–6]. The risk factors for SDB include male sex, obesity, African American ethnicity, asthma, and allergies [7]. Given the high prevalence of 4–11% [8, 9], and the negative associations, scrutiny of the pathogenesis of risk factors and effectiveness of treatment offered is of great importance. Current guidelines consider adenotonsillectomy (AT) as the first line surgical solution for pediatric SDB [10]. However, as much as 20 to 40% of patients treated for SDB with AT fail to improve, resulting in a noteworthy volume who need further treatment [11, 12].

**Fig. 1:**
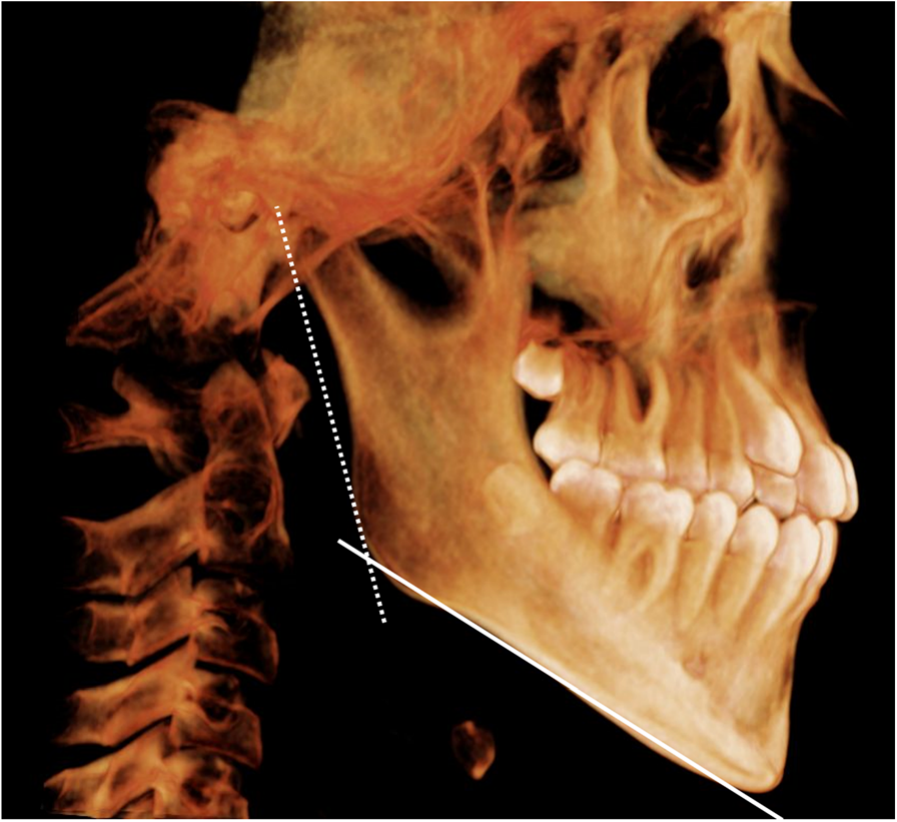
Lateral 3D image reconstructed from CBCT showing the gonial angle; formed by the mandibular plane (solid line) and ramus plane (dotted line)

Although prevalence estimates and the negative outcomes of pediatric SDB are well described within the literature, there is a paucity of data regarding predictive factors for failure of AT [13]. Previously identified independent risk factors of failure include age, obesity, chronic rhinitis, deviated nasal septum and tonsil size. We expect other patient related variables which are yet to be accounted for, or researched, to play a role. In the late 1800’s Tomes was one of the first to described an association with upper airway obstruction and morphological facial changes due to adenoid hypertrophy which he termed “adenoid facies” [14]. Since then research has supported this finding with changes in the nasal passage and gonial angle as a result of upper airway obstruction [15, 16]. The gonial angle is that formed by the mandibular plane and ramus, Fig. 1. A large gonial angle would indicate backwards (clockwise) rotation of the mandible causing the tongue/tongue base to be situated inferiorly-posteriorly and potentially cause pharyngeal airway obstruction. Furthermore, it can cause the development of anterior open bite thus promoting or enabling mouth breathing. Whereas retrognathia indicates a small jaw regardless of its angle relative to the horizontal plane. They are separate entities, possibly can occur together or independently.

On the other hand, there are two patterns of pharyngeal compromise that may occur in SDB, namely pharyngeal obstruction or collapse, and can be identified on drug induced sleep endoscopy (DISE) or imaging [17, 18]. The pharynx may exhibit collapse upon inspiration in concentrically, lateral wall to lateral wall, or at the tongue base level [17]. The significance and associations related to each type of collapse has neither been studied nor determined to date. But the proponents of DISE argue that its findings may provide an insight into the mechanisms of airway dysfunction for which there may be solutions other than traditional procedures. Therefore, we sought to conduct an exploratory study to find a phenotypic marker (gonial angle) associated with SDB in which base of tongue collapse is found upon DISE.

## Methods

### Study design

A retrospective chart review was conducted at the Stollery Children’s Hospital, Edmonton, Alberta, Canada in order to explore the relationship between the gonial angle and pharyngeal collapse at the base of tongue in children with SDB. This study received IRB ethics approval (Pro00067134). Eligible children were those assessed at the multidisciplinary Upper Airway Clinic for a combination of persistent symptoms of SDB and maxillo-mandibular disproportion (MMD) from January 2009 till July 2016. Disciplines involved in this clinic are Pediatric Orthodontics, Pulmonary medicine, and Otolaryngology- Head and Neck Surgery.

The inclusion criteria comprised ages 6–17 years old, Pediatric Sleep Questionnaire (PSQ) score over 33 [19], features of MMD requiring radiologic assessment by cone beam computerized tomography (CBCT), and having undergone DISE (and being eligible for operative treatment). All patients were scanned with the same CBCT machine in the seated-natural head position, asked to rest their tongues at the anterior teeth, and the total scan time was 4.8 s. The study group were those who exhibited tongue base collapse (TBC) on DISE, whereas the comparison group did not. We excluded children who had a history of maxillo-facial trauma or surgery, congenital craniofacial abnormalities or syndromes, and those who had incomplete data (including those with an incomplete view of the epiglottis or mandible on CBCT).

Variables were collected from electronic medical records, the electronic repository of CBCT, surgical database of the otolaryngologist and video documentation of the performed DISE. These included demographics, diagnoses, procedures performed, McGill score for overnight sleep oximetry [20], gonial angle measurement, inclination of the epiglottis on CBCT, type of pharyngeal collapse on DISE, and surgery performed.

DISE was conducted using a uniform technique and reported in a structured format that was previously validated. The patients were kept spontaneously breathing throughout the assessment in the operating room, using Remifentanyl 2–2.5 mcg/ml and infusion rates of Propofol varying from 200 to 350 mcg/kg/min titrated for response to stimulation [18]. DISE was always conducted under the same anesthetic where the surgery planned will take place.

### Measures

A certified maxillo-facial radiologist conducted two one-on-one training sessions for a senior medical student on preforming the CBCT measurements. The medical student measured the angle of the mandible (gonial angle) and inclination of the epiglottis. Prior to performing the actual measurements, an intra and inter-rater assessment on a sample (*n* = 10) of patients, not included in this pilot, demonstrated 0.95 agreement (Cohen’s kappa) between the student and the expert.

**Fig. 2:**
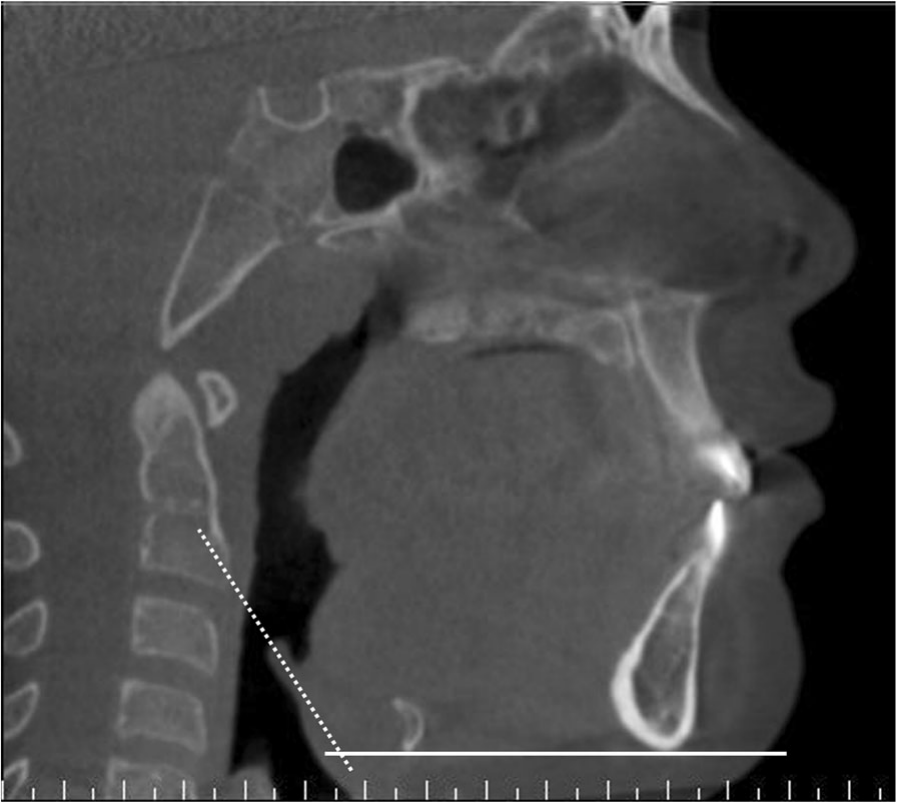
Sagittal CBCT image showing the inclination of epiglottis; the angle formed by the long axis of epiglottis (dotted line) relative to the horizontal plane (solid line)

Prior to any measurement, the CBCT volume was adjusted such that the Frankfort plane (eye-ear plane) and interorbital line were parallel to the horizontal plane, and any right-left rotations were corrected. The gonial angle, as conventionally described, was that between the intersection of the ramus and the mandibular lines (Fig. 1) [21]. The inclination of the epiglottis was measured as the angle at the intersection between the horizontal plane and a line drawn through long axis of the epiglottis (Fig. 2). The latter was chosen based on an observation by the multidisciplinary group of a possible association between large gonial angle and posterior tilt of the epiglottis, which may affect airflow into the laryngeal inlet.

The assessor of the gonial angle and the epiglottic inclination was unaware of the type of collapse in the pharynx. The subjects were recruited from a database which was intended for use in another project.

### Outcome measures

The primary outcome measure was the difference between means of gonial angles of study and comparison groups. The secondary outcome measure was the correlation between epiglottic inclination and gonial angle.

### Statistical analysis

Basic descriptive statistics were conducted to obtain the mean and standard deviation of the angle of mandible and the inclination of the epiglottis. An independent sample T-test was used to assess the statistical variance between the study and comparison gonial angles. Pearson r correlation analysis and r^2^ statistics were conducted between the angle of mandible and inclination of the epiglottis. All analyses were conducted and completed using SPSS 23.

## Results

**Fig. 3:**
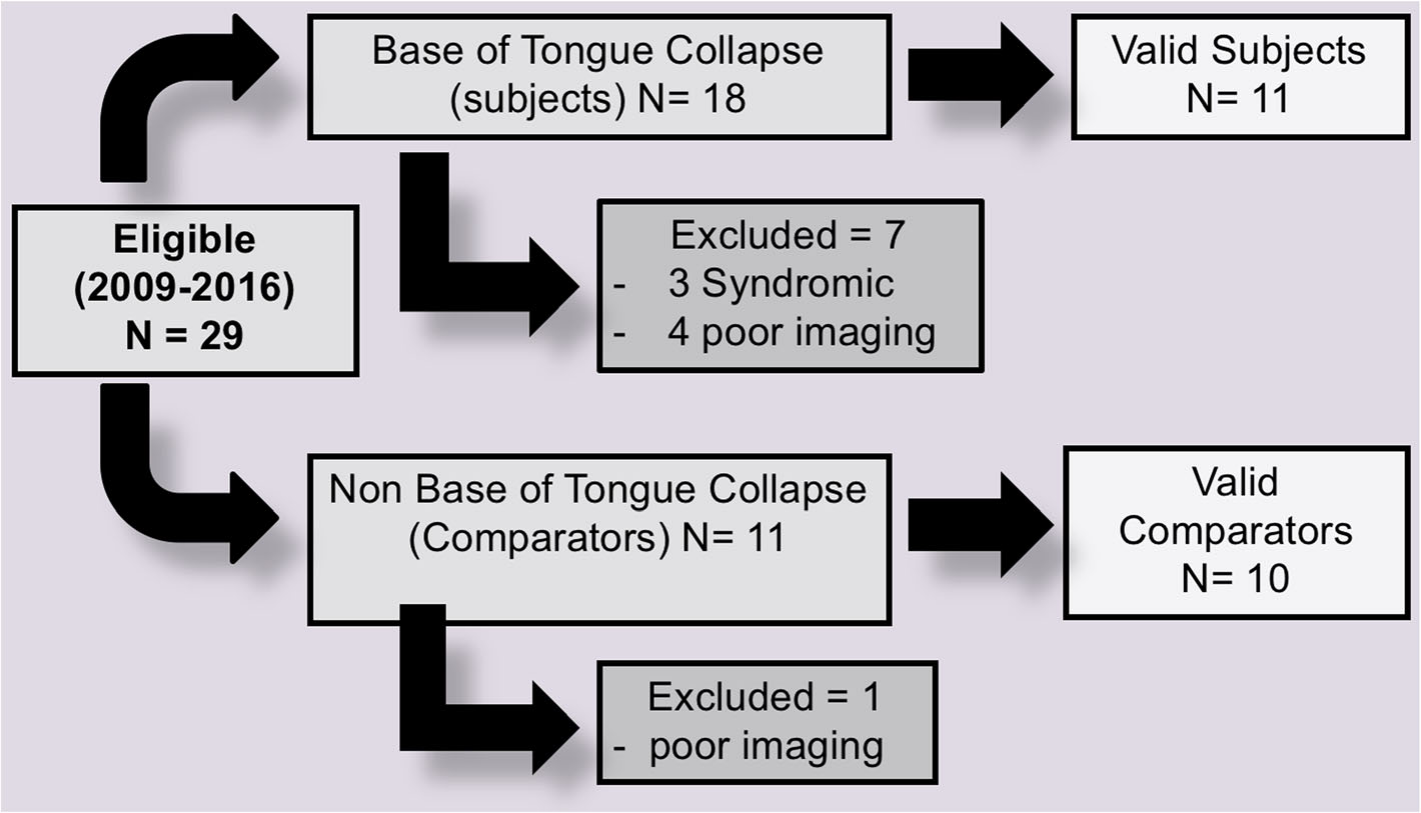
Recruitment of patients for inclusion and account for exclusions

In total 29 eligible patients, 18 male and 11 female ages 6–12 (median = 9) were identified (Fig. 1). Eighteen of whom met the inclusion criteria as study group, and 11 as the comparator group (Table 1) and Fig. 3. Seven patients out of the study group and one out of the comparator group were excluded, Fig. 2. As a result, we included 11 (seven males) valid subjects and 10 (six were males) valid comparators. In the comparison group, four patients exhibited lateral and six circumferential pharyngeal collapse upon SNP.

**Table 1:** Comparison between means of gonial angle of the study and comparison group. 95CI: 95% confidence interval

Parameter	Subjects (n of 11)	Comparison (n of 10)
Male: female	7:4	6:4
Age in years (median & range)	9 (6–12)	7 (5–12)
Comorbidities		
Chronic rhinitis	4	5
Obesity	0	1
Asthma	1	2
Surgeries performed		
Adenotonsillectomy	6	5
Tonsillectomy	1	2
Adenoidectomy	4	3

The median age for the study group was 9 years (range = 6–12) and 7 (range 6–13). for the comparison group. Comorbidities and types of surgeries are summarized in Table 1. AT was the most common surgery performed followed by adenoidectomy. There was no difference between the parameters of the sleep oximetry between the two groups (Table 2).

**Table 2:** Sleep oximetry parameters

Parameter	Subjects	Comparison
McGill Oximetry Score		
1	3 (27%)	0 (0%)
2	5 (46%)	7 (70%)
3	3 (27%)	3 (30%)
4	0 (0%)	0 (0%)
Mean Desaturation Index ± SD	6.4 ± 4.6	7.2 ± 5.5
Mean Sa02 Saturation ± SD	96.1 ± 2.2	95.7 ± 3.1
Mean Sa02 Nadir ± SD	86.2 ± 5.4	83.8 ± 6.5

The mean gonial angle of the study group were 139.3° ± 7.6° (95% CI 134.8–143.8), as opposed to 129.4° ± 3.5° (95% CI 127.2–131.6) in the comparison group which were significantly different (*p* < 0.01). The mean difference is − 9.937 (95% CI of − 15.454 to − 4.421, *P* = 0.001). The effect size is 1.675 and is large. Since the study group values were not normally distributed, Mann Whitney test was also run and the difference between the medians was still significant. However, the mean difference between the epiglottic inclination in the study (123.0° ± 4.6°) and the comparison groups (121.45° ± 6.94°) was 1.932 (95% CI -3.389 to 7.252, *P* = 0.457), which was not significant, but the test was short of the recommended power of 0.8 (0.05).

Analysis of our secondary outcome showed that within the whole cohort, inclination of the epiglottis had a mild positive correlation (*r* = 0.32, *p* < 0.05) between gonial angle and inclination of the epiglottis.

## Discussion

Current recommended first line surgical treatment for SDB is AT [10]. However, given the significant proportion of patients who fail to receive benefit from such treatment, evidence providing insight into predictors of the pathophysiology of patients SDB could reduce unnecessary and ineffective surgical procedures. Gonial angle, was chosen based on the clinical observation of the interdisciplinary airway team during the six years’ experience in the clinical and radiographic assessment of children with MMD and Snoring/Sleep Disordered Breathing.

Our exploratory study demonstrated that the children with SDB and MMD who exhibited TBC has a larger mean gonial angle (139.3° ± =7.6°), than their counterparts who exhibit different types of collapse (129.4° ± 3.5°), and more than the reported mean values of gonial angle for this age group in the literature (133.96° ± =7.6°) [21]. A mild positive correlation (*r* = 0.32, *p* < 0.05) was also found between the size of the gonial angle and the inclination or posterior tilt of the epiglottis. This suggests that downward growth of the mandible might be associated with that particular type of pharyngeal tongue collapse.

This is the first study to investigate how dynamic endoscopic findings during chemically induced sleep can relate to static 3D imaging (CBCT) in children with SDB. There is a surging interest into the role of imaging in assessing SDB in children and specifically in orthodontic and maxillofacial literature [18]. In the adult literature, a systematic review was conducted to assess the most important anatomical characteristics of the upper airway related to the pathogenesis of obstructive sleep apnea by analyzing the three-dimensional parameters (using different imaging modalities) of the airway column. The minimum cross-sectional area was the only one that was reduced consistently in obstructive sleep apnea (OSA) patients. The majority of the studies were of fair quality and quite heterogenous precluding a meta-analysis [22]. Another systematic review, this time in the pediatric literature [23], indicated that multiple cepahlometric measures of children diagnosed with MMD are statistically different between those who were asymptomatic and those with primary snoring and/or SDB. However, given the modest differences demonstrated, the clinical significance was deemed questionable. Alsufyani et al. [18] in a review of the literature indicated that several modalities had been used to perform three-dimensional analysis and measurements of the airway of SDB patients before and after treatment, including magnetic resonance imaging, multi-detector computed tomography and CBCT. The group commented that aside from the well known inherent advantages and disadvantages of each modality (expense, radiation exposure, physics inadequacies) the authors highlighted the challenges remaining with respect to image acquisition, three-dimensional reconstruction and analysis. Thus far nearly all the work concentrates on static measures (be them two dimensional of volume related) and how they relate to cross sectional polysomnographic parameters and their changes after various treatment lines.

Some of this work is in support of our observation. For example, Finkelstein et al. [24], in 2000 studied a group of children with nasal obstruction and counterparts without, all of whom did not have tonsillar enlargement and were otherwise healthy. They explored the relation between cephalometric measures and the severity of their symptoms (5 grade severity ranging from none to nasal obstruction with universally observed snoring and obstructive symptoms during sleep – but no objective or validated sleep parameters). An increased gonial angle was associated with increased symptoms which was statistically significant, and interestingly within the values which we reported and more than those reported for normative values, although including wider age group than ours. In another clinical study by Iwasaki and coinvestigator [25], after treating 28 subjects with rapid maxillary expansion, a significant increase in pharyngeal airway volume was demonstrated which they related to improvement in tongue position.

However, the study that may lend support to our work was done by Watanabe and colleagues [26]. In a study of the impact of body habitus and craniofacial parameters on pharyngeal closing pressures, SDB adult patients were found to have receded mandibles, with longer faces and downward mandibular growth. The angle reflecting the development of the mandible was significantly different between SDB and normal patients (mean of 36.0° range of 34.0°– 41.5° versus a mean of 30.5° and range of 27.0°–33.0°, *p* < 0.05). This study correlated cephalometric, two-dimensional measures with a dynamic assessment of pharyngeal collapse, along the same lines of the current work.

Further epidemiological and biological studies are warranted to confirm this association and to provide further insight into biological mechanisms for purported association. The concept of measuring a dynamic soft tissue structure in a static cone beam CT is far from accurate. However, we attempted to standardize patient posture and use small scan time thus reducing chances of motion or multiple breathing cycles. We are aware of our small sample size, and the lack of polysomnographic data which prevents us from making any generalizable conclusions to the larger population, nor establishing a dose response relation. However, given this is an exploratory study, and the first to investigate the correlation between gonial angle, the static inclination of the epiglottis and dynamic TBC in children with SDB, findings are valuable for hypothesis generation for future larger scale studies.

## Conclusions

The preliminary observation indicates that tongue base collapse is associated with a large gonial angle, which provides a hypothesis for a phenotypic marker that could explain persistence of SDB after traditional AT.

## Abbreviations

CBCT: Cone beam computed tomography
DISE: Drug induced sleep endoscopy
MMD: Maxillo-mandibular disproportion
OSA: Obstructive sleep apnea
PSQ: Pediatric Sleep Questionnaire
SDB: Sleep disordered breathing
SNP: Sleep nasopharyngoscopy
TA: Adenotonsillectomy
TBC: Tongue base collapse
